# Reevaluating BED in cervical cancer HDR brachytherapy: source decay and tissue-specific repair significantly impact radiobiological dose

**DOI:** 10.3389/fonc.2025.1407606

**Published:** 2025-05-05

**Authors:** Suyan Bi, Jiaomei Zhou, Meiling Xu, Zhitao Dai

**Affiliations:** ^1^ School of Medical Sciences, Universiti Sains Malaysia, Kelantan, Malaysia; ^2^ National Cancer Center/National Clinical Research Center for Cancer/Cancer Hospital & Shenzhen Hospital, Chinese Academy of Medical Sciences and Peking Union Medical College, Shenzhen, China

**Keywords:** high-dose-rate brachytherapy (HDR BT), cervical cancer, source decay impact, tumor cell lines, intrafraction damage repair, biological effective dose (BED)

## Abstract

**Objective:**

This study aims to investigate the influence of intrafraction DNA damage repair on biologically effective dose (BED) in Ir-192 high-dose-rate (HDR) brachytherapy (BT) for cervical cancer. Specifically, we examine BED variations resulting from source decay at various treatment time points across different tumor cell lines and normal tissues.

**Methods:**

Instead of the simplified BED formula, which does not account for intrafraction and interfraction repair or tumor repopulation, we applied the generalized BED (*BEDg*) formula. BED values for various subtypes of cervical cancer tissues and Organs at Risk (OARs) were calculated using both BED formulas across a full source exchange cycle.

**Results:**

The results demonstrate that *BEDg* values are significantly lower and decrease more markedly and extended treatment time compared to *BED* values. For tumors with *α/β* = 10, the maximum BED deviation (Δ*BED* = *BED* − *BEDg*) reached 3.05% ± 0.47% at *D*_90%_ of the High-Risk Clinical Tumor Volume (HRCTV) in BT. For specific cervical cancer subtypes, the three largest Δ*BED* (%) values at *D*_90%_ of HRCTV were 14.06 ± 1.67 (stages I–II, *α/β* = 10), 9.92 ± 1.19 (HX156c, *α/β* = 16.46), and 7.57 ± 1.05 (HX155c, *α/β* = 11.40). Similar trends were observed in OARs. As the source decays, the maximum Δ*BED* (%) at *D*_0_._1_ *_cc_* was 13.37 ± 2.27 (bladder), 11.92 ± 2.10 (rectum), 12.45 ± 2.27 (sigmoid), and 11.91 ± 2.62 (small intestine), assuming *α/β* = 3.

**Conclusions:**

These findings confirm that source decay significantly impacts BED in cervical cancer treatment, affecting both tumor tissues with varying radiosensitivities and normal tissues. The simplified BED formula tends to overestimate the actual dose, especially at a source activity of 2 Ci, highlighting the necessity of using the full *BEDg* model for accurate dosimetric evaluation in HDR brachytherapy.

## 1 Introduction

Cervical cancer is a highly prevalent form of malignancy among women worldwide, particularly in developing countries (1, 2). Brachytherapy (BT) is an indispensable component of the treatment paradigm for cervical cancer because it enables comprehensive tumor coverage while concurrently minimizing radiation exposure to adjacent healthy tissues (3). When integrated with external beam radiation therapy (EBRT) and platinum-based chemotherapy, brachytherapy serves as the cornerstone of treatment for locally advanced cervical cancer and significantly augments the overall survival rates of affected patients (4–6). Currently, high-dose rate (HDR) brachytherapy has gained widespread adoption in numerous hospitals. It employs a single approximate point source to mimic a line source for targeted volume irradiation, a technique tailored to optimize tumor-specific radiation delivery. In cervical cancer BT, dosage delivery schemes are meticulously designed based on their association with radiotoxicity to critical organs such as the vaginal surface, bladder, small intestine, and rectum (7). Typically, in an HDR BT treatment plan, a prescription dose of 5.5 Gy–7 Gy is administered twice a week to the high-risk clinical tumor volume (HRCTV), ensuring that 90% of the volume (*D*_90%_) of the HRCTV receives the prescribed dose accurately. A total of three to five fractions are delivered either in conjunction with or without concurrent chemotherapy (8, 9).

Dimopoulos et al. (10) conducted an in-depth analysis of the correlation between the dose-volume histogram (DVH) and local tumor control. Their findings revealed that a *D*_90%_ *>* 85 Gy for HRCTV was associated with remarkable local control in the absence of chemotherapy. The dose accumulation of BT and EBRT was converted into the equivalent dose in 2 Gy (*EQD*_2_) using the linear-quadratic (LQ) model with an *α/β* value that is uniquely determined by the tumor type, as illustrated by Madan et al. (11). The radioactive isotope Ir-192 is commonly used as a radiation source in brachytherapy (12, 13). The radiation dose for HDR BT is typically computed using the formalism detailed in the updated American Association of Physicists in Medicine (AAPM) Task Group No. 43 report, which provides the essential parameters for accurate dose distribution calculation (14). Given the inherent variations in source activity during a source-exchange cycle, the decay of the source must be meticulously considered when accounting for differences in treatment time. In a three-dimensional (3D) BT treatment planning system (TPS), this difference can be automatically calculated because of the exponential decay of source strength over time (13, 15). Otani et al. (13) and Demanes and Ghilezan (16) revealed that treatment times vary substantially depending on the source activity during the source-exchange cycle, with delivery times often exceeding 30 min for single-fraction regimens. When DNA damage repair mechanisms are factored into, these protracted dose-delivery times have a profound impact on the biologically effective dose (BED), which is calculated using the LQ model, as corroborated by multiple studies (17, 18). For instance, in prostate cancer, when the extended time exceeds the half-life for DNA damage repair in prostate cancer cells, the biological effectiveness of the delivered dose can be significantly attenuated (19). In fact, the current equivalence metrics used in clinical practice to compare different dose-delivery schemes for cervical cancer were analyzed and developed based on a simplified BED equation that failed to incorporate the effects of intrafraction DNA damage repair and cell repopulation (16).

It is well established that cells exhibit diverse DNA damage-repair times. Investigating the biological effects of various tumor cell subtypes at the clinical level is of utmost importance in cervical cancer radiotherapy (20, 21). In recent years, researchers have focused on the radiobiological effects of various cervical cancer cell lines. The BED characteristics of numerous cell lines were determined by *in vitro* studies of the irradiated isolated cells. These studies revealed that different cell lines display varying sensitivities to radiation, leading to distinct *α/β* values. For instance, Chow et al. (20) discovered that in cervical cancer cell lines such as CaSki, C33A, SiHa, and SW756, the measured *α/β* ratios were consistently lower (5.2 Gy, 5.6 Gy, 6.3 Gy, and 5.3 Gy, respectively) than the conventionally accepted values in clinical practice (*α/β* = 10 Gy, $T_{1/2}=1.5\;\text{h}$ ), while the $T_{1/2}$ values were higher (3.3 h, 2.7 h, 2.8 h, and 4.8 h). However, in routine clinical practice, the implications of different BEDs resulting from the variable radiation sensitivities of cell lines have not been adequately considered.

Consequently, the objective of this study was to utilize the full-form of BED, which incorporates DNA damage repair and cell repopulation factors, to comprehensively evaluate the extent of BED variation caused by source decay in cervical cancer patients with diverse cell lines. Additionally, we aimed to establish the clinical significance of this effect in the context of cervical cancer treatment using BT.

## 2 Materials and methods

### 2.1 Case selection

A retrospective analysis was performed on patients with cervical carcinoma (FIGO stages III–IV) who were treated at Shenzhen Cancer Hospital between January 2019 and February 2020 and had not undergone prior surgery. A total of 24 patients received EBRT with 45 Gy in 25 fractions, encompassing the entire primary tumor and associated lymphatic drainage area. For these patients, brachytherapy (BT) with a classical dosimetric model was selected as the treatment approach. The treatment regimen involved administration of 5 Gy–9 Gy per fraction twice a week for 2 weeks. The initiation of BT was determined based on the clinical response evaluated by the radiation oncologist, either starting in the third week of EBRT or after the completion of EBRT, as suggested by Gill et al. (22) and Weitmann et al. (23). Chemotherapy was not administered on the same day as BT to avoid potential toxicity concerns, as advised by Gill et al. (22).

During BT, a set of applicators (Varian, Manchester System) was carefully placed in the patients’ bodies under anesthesia. After treatment, the applicators were removed. Intracavitary HDR Iridium-192 BT was carried out using oval-shaped and tandem applicators in conjunction with the BT dose-delivery device. Radiation oncologists utilized computed tomography (CT) images to verify the accurate positioning of the applicators and delineate the high-risk clinical tumor volume (HRCTV) and organs at risk (OARs). Magnetic resonance imaging (MRI) was also employed to precisely determine the boundaries of the HRCTV.

### 2.2 Planning design and optimization

Applicator reconstruction and treatment planning design were conducted on the CT images using the Eclipse*™* Brachytherapy Planning System (Varian Medical Systems, Palo Alto, CA, USA, version 13.6). Standard treatment plans were formulated based on reference Point A defined according to the anatomical structures in CT images, as described by Rivard et al. (14, 24), along with standard source-loading patterns, dwell positions, and weights. The HRCTV and OARs, including the rectum, bladder, sigmoid, and small intestine, were contoured and incorporated into the optimization process. The optimization process entailed meticulously adjusting the source positions and dwell times manually, as well as manipulating isodose lines, to ensure that the dose-volume histograms (DVHs) of both the HRCTV and OARs met the prescription constraints. In addition, meticulous attention was paid to dose distribution to prevent the introduction of excessive heterogeneity. During routine clinical treatment, dose calculations and reports were based on the total biologically equivalent dose in 2 Gy/fraction (EQD_2_), encompassing both EBRT and BT. The LQ model for radiation damage repair was utilized with *α/β* ratios of 10 Gy for tumors and 3 Gy for OARs (25). The goal of combining EBRT and BT is to deliver a minimum total dose of 84 Gy to at least 90% of the HRCTV volume. A dose constraint of 90 Gy (*D*_2_ *_cc_*) was applied to the bladder, whereas constraints of 75 Gy (*D*_2_ *_cc_*) were imposed on the rectum, sigmoid colon, and small intestine. The clinical prescription dose was 7 Gy–8 Gy × 4 fractions for *D*_90%_ of the HRCTV, and the dose limits are presented in **Table 1**.

**Table 1:** Total dose limits of BT and EBRT in terms of EQD_2_.

ROIs	Metrics	*α/β* (Gy)	Total EQD2 (Gy)		d (Gy)	
			1st obj.	2nd obj.	1st obj.	2nd obj.
HRCTV	D_90%_	10	≥84	–	7.01	–
Bladder	D_2_ *_cc_*	3	≤90	80	6.29	5.45
Rectum	D_2_ *_cc_*	3	≤75	65	4.98	3.93
Sigmoid	D_2_ *_cc_*	3	≤75	65	4.98	3.93
Intestine	D_2_ *_cc_*	3	≤75	65	4.98	3.93

### 2.3 The linear quadratic model

In the conventional calculation of the biologically effective dose (BED, in Gy) for brachytherapy, when ignoring DNA damage repair and cellular repopulation, the simplified BED model for a total dose *D* (in Gy) delivered in *n* fractions with a dose per fraction *d* (in Gy) is given by Rivard et al. (14) and Weitmann et al. (23):


(1) $$BED=D\left(1+\frac{d}{\alpha /\beta }\right)$$

To investigate the influence of intrafraction DNA damage repair and cellular repopulation on HDR brachytherapy regimens, a full linear-quadratic (LQ) cell survival model or the derived concept of full-form BED (*BEDg*) was employed in this study. *BEDg* (in Gy) was provided by Curtis (26) and Fowler et al. (18):


(2) $$BEDg=D\left(1+\frac{g}{\alpha /\beta }d\right)-\frac{ln\left(2\right)}{\alpha T_{d}}\left(T-T_{k}\right)$$

where parameters *D*, *d*, *α*, and *β* are the same as those in Equation 1. Parameter *g* is the time-protraction factor that accounts for the effect of DNA damage repair during the delivery of a single fraction (in the simple BED, *g* = 1). *T_d_* is the effective tumor doubling time, which represents cellular repopulation (in the simple BED, *T_d_* = ∞). *T* is the total elapsed time of the treatment course, and *T_k_* is the onset or lag time of the cell repopulation. If *T <T_k_*, then the second term in Equation 2 is equal to 0.

The dose protraction factor *g* in Equation 2 was used to describe the effect of intrafraction on DNA damage repair. Given the 73.81 day half-life of Ir-192, the dose rate during delivery of one treatment fraction smaller than 1 h, can be considered nearly constant. Under this condition and assuming mono-exponential repair kinetics, the dose-protraction factor *g* is given by Curtis (26) and Fowler et al. (18):


(3) $$g=\frac{2}{{\left(\mu t\right)}^{2}}\left(e^{\mu t}+\mu t-1\right)$$

where *t* denotes the duration of dose delivery for a given dose fraction, the DNA repair rate $\mu \frac{ln\left(2\right)}{T_{1/2}}$ , and $T_{1/2}$ is the DNA damage repair halftime. Overall, the magnitude of *g* depends on the repair rate and the duration of dose delivery. The value of *g* ranges from a minimum of 0 (instant repair, *i.e.*, $T_{1/2}\to 0$ ) to a maximum of 1 (no repair, *i.e.*, $T_{1/2}\to \infty$ ).

The cellular repopulation process is particularly crucial during extended treatment courses, such as those lasting one or two months. This can lead to a reduction in the effective dose delivered to the tumor, as captured by the second term in Equation 2. Four key factors have a positive impact on cellular repopulation during radiotherapy: an extended overall treatment course *T*, faster onset of proliferation (smaller *T_k_*), reduced radiosensitivity (smaller *α*), and faster tumor cell proliferation (shorter *T_d_*). The treatment course for BT in this study consisted of twice-weekly sessions with a total of 4 fractions, resulting in a treatment duration of approximately 14 days. Usually, *T_k_* = 17–31 is applied, according to the literature (27, 28). Consequently, the second term in Equation 2 equals to 0 in this study.

**Figure 1:**
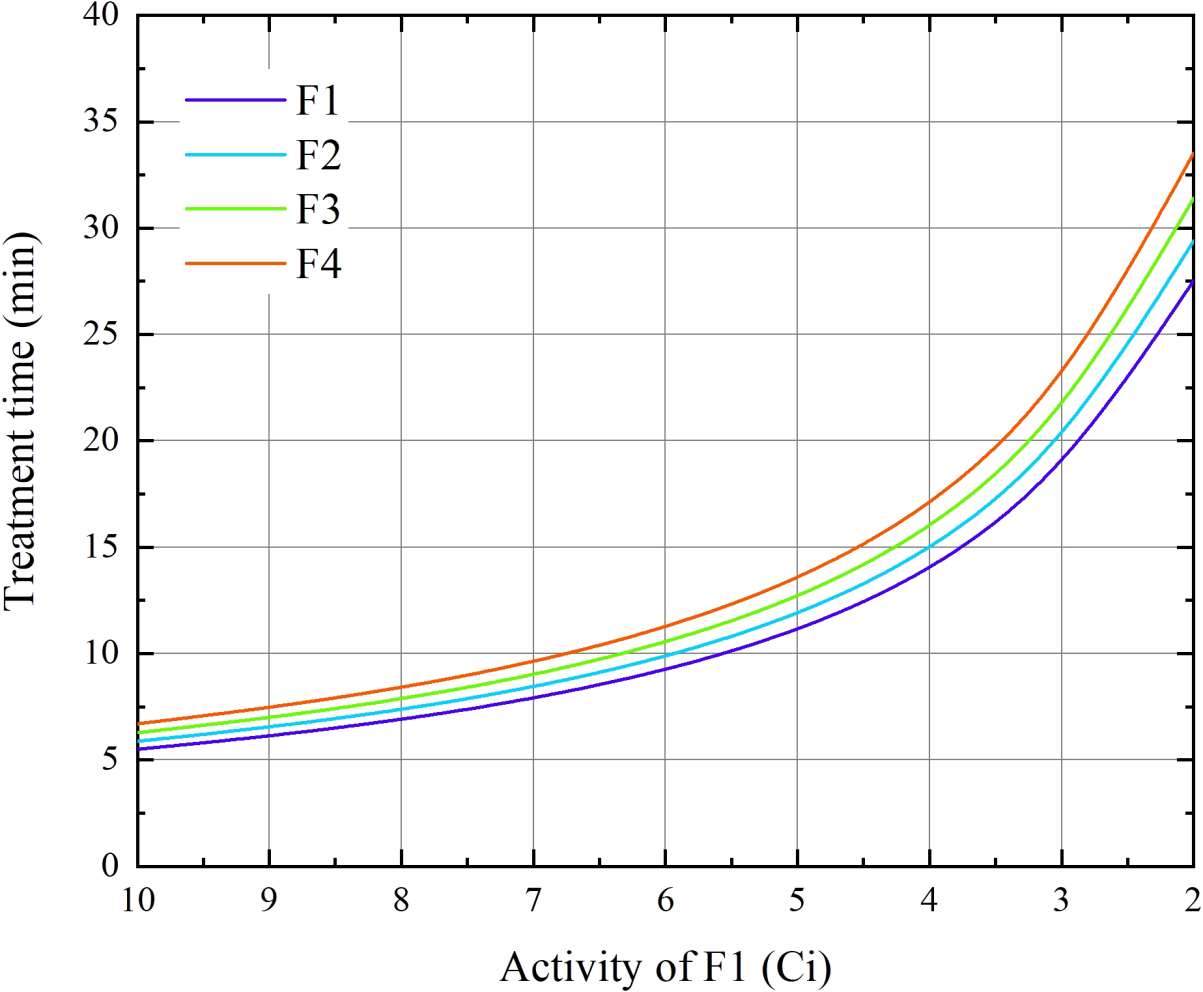
The duration of BT treatment for the four fractions as functions of the source activity at the 1st fraction for 24 cervical patients.

The treatment times of the first fraction (*t*_0_) for each case in the were obtained from the treatment planning system (TPS). This time represents the nominal time, assuming a source activity of *A*_0_ = 10 Ci. The treatment time *t* for different activity *A* can be calculated according to the equation *At* = *A*_0_ *t*_0_ (as shown in **Figure 1**). In this study, we assumed a time interval between fractions of 3.5 days. For the treatment times of the remaining three fractions, we first calculated the decay activity at the corresponding time of each fraction according to the decay function of Ir-192 and then determined the treatment time using the equation *At* = *A*_0_ *t*_0_.

### 2.4 Radiobiological parameters chosen

In accordance with the AAPM and ICRU report, *α/β* = 3 Gy and *T*_1_ *_/_*_2_ = 0.5 were recommended for OARs, while *α/β* = 10 Gy and $T_{1/2}=1.5$ h were recommended for tumors (14, 29); ICR (30). Additionally, based on clinical trials for stage I and II cervix carcinoma, parameters with *α/β* = 52.63 Gy and 10 Gy, with $T_{1/2}=1.5$ and 0.25 h were reported, respectively (31). Regarding cervical cancer cell lines, Kelland and Steel (21) and Chow et al. (20) discovered that the nine cell lines they studied had unique *α/β* and $T_{1/2}$ values. The parameters are listed in **Table 2**.

**Table 2:** Model parameters of OARs and different cell lines of cervical cancer taken from literature.

Cell lines	α/β	$T_{1/2}\;$ (h)	Reference
Conventional recommendation (OAR)	3.00	0.50	ICRU Report 89ICR (30)
Conventional recommendation (tumor)	10.00	1.50	ICRU Report 89ICR (30)
Stage I and II cervix carcinoma	52.63 10.00	1.50 0.25	Roberts et al. (31)
HX151c	11.46	1.90	Kelland and Steel (21)
HX155c	11.40	0.50	
HX156c	16.46	0.26	
HX160c	6.01	5.70	
HX171c	7.02	2.30	
CaSki	5.20	3.30	
C-33A	5.60	2.70	Chow et al. (20)
SiHa	6.30	2.80	
SW756	5.30	4.80	

In this study, we utilized the full-form BED formula to calculate *BEDg* values under different fractionation schemes, considering the physical doses at various source activities. This involved considering the transition from a new source of 10 Ci to an old source of 2 Ci during a source exchange cycle. The BED values of the HRCTV and OARs were calculated for each patient using both simplified and full-form BED formulas. In addition, based on the corresponding *α/β* values for different cell lines, we calculated the BEDg values of the HRCTV for each case in all cell lines. These values were recorded and subjected to statistical analysis. Additionally, the potential clinical implications of these findings are discussed.

### 2.5 Statistical analysis

Statistical analyses were conducted, and figures were created using SPSS25.0 software (IBM), Origin software (NY, USA), and Microsoft Office 2022. The *χ* ^2^ test and Student’s t-test were used to assess the compositional ratio differences between the two groups. Statistical significance was set at *p <*0.05.

## 3 Results

### 3.1 Statistics of physical metrics

**Figure 2:**
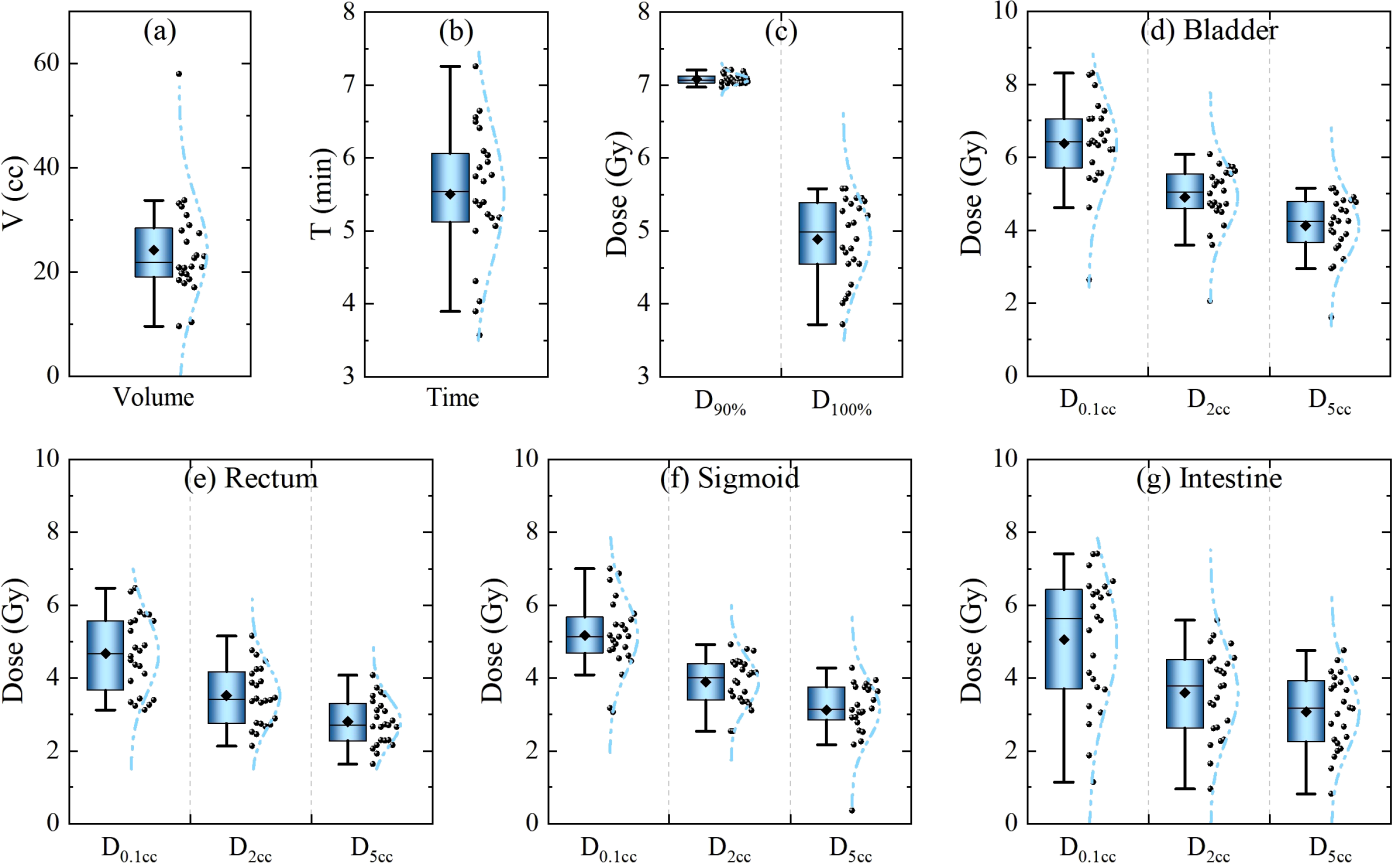
Statistics of physical metrics of the 1st fraction of 24 cervical cancer patients. **(a)** Target volume and **(b)** treatment duration. **(c–g)** Dosimetric parameters for the target volume, bladder, rectum, sigmoid, and intestine.

The physical metrics of 24 cervical cancer patients were comprehensively analyzed. Specifically, the volumes of HRCTV, treatment times, and physical dose of HRCTV and Organs at Risk (OARs) in the first fraction were accurately recorded, as displayed in **Figure 2**). The volume of HRCTV, as depicted in **Figure 2a** ranged from 9.5 cc to 58 cc, with a median value of 21.8 cc. The average treatment time was 5.5, spanning from 3.5 min to 7.3 min. **Figures 2c–g** display the dosimetric parameters for the target volume, bladder, rectum, sigmoid, and intestine. Dosimetric metrics, including *D*_90%_ and *D*_100%_ HRCTV, as well as *D*_0_._1_ *_cc_*, *D*_2_ *_cc_*, and *D*_5_ *_cc_* for OARs, were precisely obtained. Notably, all of these physical indicators met the strict dose limits listed in **Table 1**, indicating the feasibility and reliability of the treatment plans in this study. This set of data not only provides a baseline for understanding the physical characteristics of the patients in this study but also serves as a crucial reference for subsequent analysis of the impact of source decay on the biologically effective dose.

### 3.2 Influence of intrafraction DNA damage repair

The influence of intrafraction DNA damage repair is a key aspect explored in this study, and it is closely related to the repair halftime and dose delivery time (as described by Equation 3). The treatment time increased in accordance with an inverse proportional function as the radioactivity of the source decreased (as shown in **Figure 1**). Correspondingly, the dose protraction factor *g* continuously decreases.

Typically, when the radioactivity of the radiation source is less than 2 Ci, it is replaced by a new one. This implies that, when delivering the same physical dose, the treatment time at the end of the source’s lifespan is five times longer than that at the beginning of a new source. In extreme cases, compared with a new 10-Ci source, the dose protraction factor *g* for single doses was reduced to 0.818 (*α/β* = 3 Gy) and 0.933 (*α/β* = 10 Gy). Considering all cell lines, the value of *g* ranged from 0.923 to 0.693 (*α/β* = 16.46 Gy, $T_{1/2}=0.26\;\text{h}$ ) within the source-exchange cycle, as presented in **Table 3**. These results are of great significance, as they quantitatively illustrate the impact of source decay on the intrafraction DNA damage repair process, which has important implications for accurately calculating the biologically effective dose in cervical cancer radiotherapy.

**Table 3:** The average *g* factors of the 1st fraction for OARs and tumors with different source activity.

Cell line	Source Activity (Ci)								
	10	9	8	7	6	5	4	3	2
Conventional OAR	0.959	0.955	0.949	0.942	0.933	0.920	0.902	0.873	0.818
Conventional tumor	0.986	0.984	0.983	0.980	0.977	0.972	0.966	0.955	0.933
Stage I and II cervix carcinoma	0.986 0.920	0.984 0.912	0.983 0.902	0.980 0.889	0.977 0.873	0.972 0.850	0.966 0.818	0.955 0.769	0.933 0.684
HX151c	0.989	0.988	0.986	0.984	0.982	0.978	0.973	0.964	0.947
HX155c	0.959	0.955	0.949	0.942	0.933	0.920	0.902	0.873	0.818
HX156c	0.923	0.915	0.906	0.893	0.877	0.855	0.824	0.777	0.693
HX160c	0.996	0.996	0.995	0.995	0.994	0.993	0.991	0.988	0.982
HX171c	0.991	0.990	0.989	0.987	0.985	0.982	0.977	0.970	0.956
CaSki	0.994	0.993	0.992	0.991	0.989	0.987	0.984	0.979	0.969
C-33A	0.992	0.991	0.990	0.989	0.987	0.984	0.981	0.974	0.962
SiHa	0.992	0.992	0.991	0.989	0.988	0.985	0.981	0.975	0.963
SW756	0.996	0.995	0.995	0.994	0.993	0.991	0.989	0.985	0.978

### 3.3 The dependence of BED and BEDg on different fraction dose and source activity

**Figure 3:**
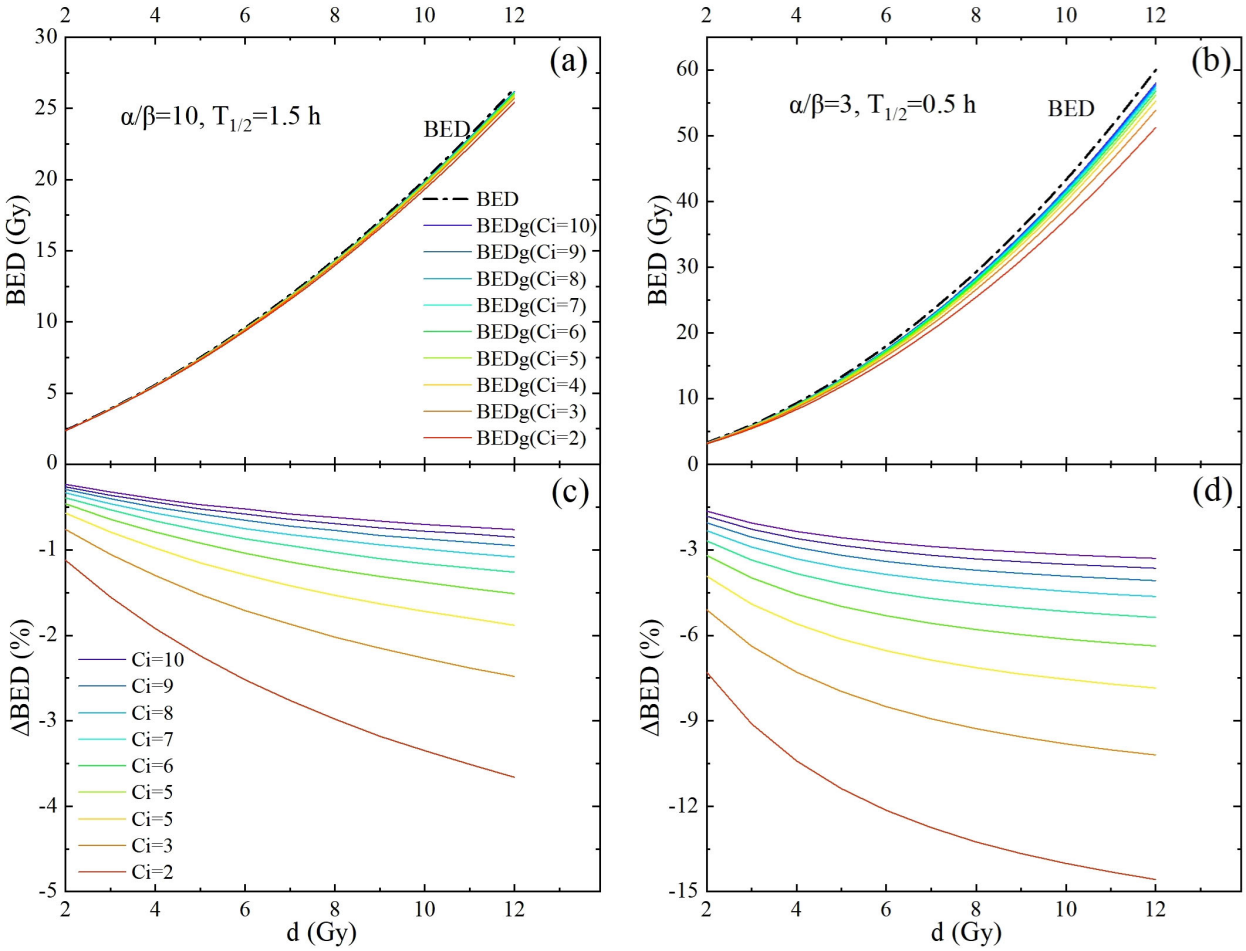
The potential impact of intrafraction repair and variation of source strength is demonstrated by performing full-form BED formula calculations (colored lines) and comparing them with simplified BED formula calculations (dash-dotted lines). This was carried out for a one-fraction scheme, with the fraction dose *d* ranging from 2 Gy to 12 Gy. **(a, b)** are BED calculated using the conventional recommendation parameters for the tumor and OARs, respectively. **(c, d)** are the differences between the BEDs calculated using the full-form and simplified BED formulas for tumors and OARs, respectively.

**Figure 4:**
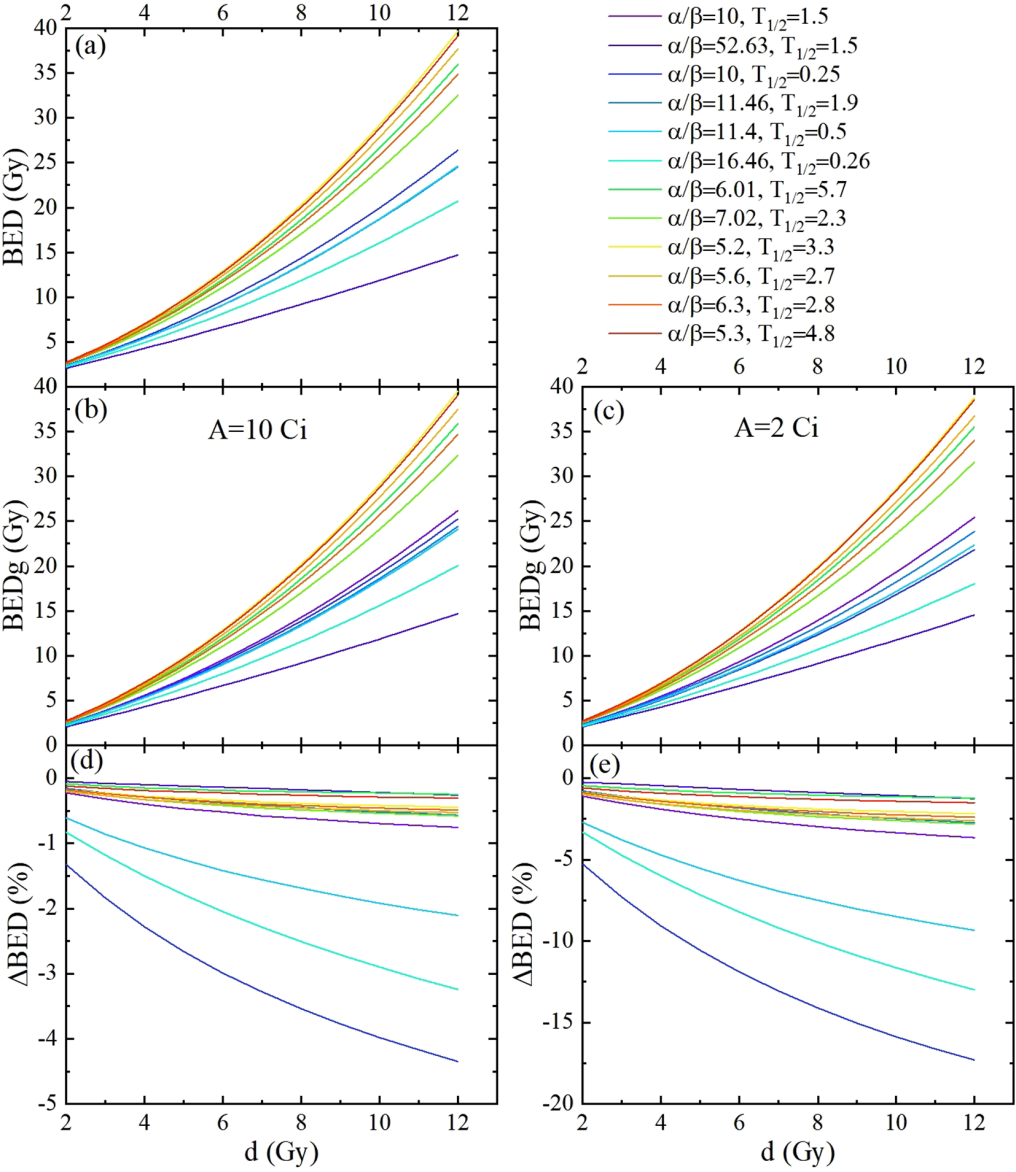
The BED and BEDg of the tumor calculated based on the model parameters listed in **Table 2**, as functions of the fraction dose. This was carried out for 1 fraction schemes, with fraction dose *d* ranging from 2 Gy to 12 Gy. **(a)** Displayed BED calculated using the simplified BED formula. **(b, c)** are the BED calculated using the full-form BED formula with source activities of 10 Gy and 2 Gy, respectively. **(d, e)** are the differences between the BEDs calculated with the full-form and simplified BED formulas with source activities of 10 Gy and 2 Gy, respectively.

**Figures 3**, **4** show novel and important findings regarding the changes in BED and BEDg in relation to the physical dose under different source activities. In the conventional recommendation, with *α/β* = 10 Gy and $T_{1/2}=1.5$ h for the tumor and *α/β* = 3 Gy and $T_{1/2}=0.5$ h for normal tissues (**Figure 3**), a significant trend was observed: the Δ*BED* values increased as the single physical dose increased. When the source activity decayed from 10 Ci to 2 Ci, the Δ*BED* reached approximately 1.9% and 7.4% for *α/β* = 10 Gy and *α/β* = 3 Gy, respectively, when the single physical dose reached 7 Gy–8 Gy. This finding highlights the importance of considering the source decay and fraction dose simultaneously in radiotherapy planning.

Considering the assumed model parameter values in **Figure 4**, which include two clinical categories (31) and nine cell lines (**Table 2**) of cervical cancer types studied in this research. Similar results were obtained; compared to BED, BEDg decreased significantly according to the fraction doses at 10 Ci and 2 Ci, respectively. The Δ*BED* values from a maximum of approximately −0.1% (carcinoma:*α/β* = 52.63 Gy, $T_{1/2}=1.5\;\text{h}$ ) to a minimum of almost −18% (carcinoma:*α/β* = 10.00 Gy, $T_{1/2}=0.25\;\text{h}$ ).These results not only confirm the significant impact of source decay on BED, but also reveal the differences in radiosensitivity among different cell subtypes, which enriches the understanding of the radiobiological mechanisms in cervical cancer radiotherapy and provides a basis for personalized treatment.

### 3.4 BED and BEDg of HRCTV dependence on assumed model parameter values

As clearly presented in **Table 4** and **Figure 4**, in the clinical categories (named cervix carcinoma) with source decay (from 10 Ci to 2 Ci), BEDg (Gy) values in *D*_90%_ and *D*_100%_ of HRCTV showed distinct changes. For *α/β* = 52.63, BEDg values ranged from 32.14 ± 0.34 to 32.02 ± 0.34 and 21.39 ± 2.56 to 21.33 ± 2.55 in *D*_90%_ and *D*_100%_ of HRCTV, respectively. For *α/β* = 10, the BEDg values ranged from 46.64 ± 0.69 to 41.59 ± 1.02 and 28.38 ± 4.08 to 25.96 ± 3.65 in *D*_90%_ and *D*_100%_ of HRCTV, respectively. These results demonstrate the significant influence of source decay on BEDg values under different *α/β* ratios, which is a new discovery in the study of cervical cancer radiotherapy dosimetry.

**Table 4:** The BED and BEDg of tumor calculated based on the model parameters listed in **Table 2** under the conditions of different source activity.

Cell line	*α/β*	${\text{T}}_{1/2}$	Metrics	BED	Source Activity (Ci)					
					10	5	2	10	5	2
						BEDg (Gy)			ΔBED (%)	
Conventional	10.00	1.50	D_90%_	48.39 ± 0.64	48.08 ± 0.64	47.78 ± 0.65	46.91 ± 0.68	−0.64 ± 0.10	−1.27 ± 0.20	−3.05 ± 0.47
			D_100%_	29.22 ± 4.23	29.07 ± 4.20	28.93 ± 4.17	28.51 ± 4.10	−0.50 ± 0.08	−1.00 ± 0.16	−2.41 ± 0.38
Cervix carcinoma	52.63	1.50	D_90%_	32.14 ± 0.34	32.14 ± 0.34	32.08 ± 0.34	32.02 ± 0.34	−0.18 ± 0.03	−0.36 ± 0.06	−0.87 ± 0.13
			D_100%_	21.39 ± 2.56	21.39 ± 2.56	21.36 ± 2.55	21.33 ± 2.55	−0.13 ± 0.02	−0.26 ± 0.04	−0.62 ± 0.11
	10.00	0.25	D_90%_	48.39 ± 0.64	46.64 ± 0.69	45.11 ± 0.79	41.59 ± 1.02	−3.62 ± 0.55	−6.77 ± 0.97	−14.06 ± 1.67
			D_100%_	29.22 ± 4.23	28.38 ± 4.08	27.65 ± 3.95	25.96 ± 3.65	−2.85 ± 0.45	−5.33 ± 0.80	−11.08 ± 1.43
HX151c	11.46	1.90	D_90%_	45.83 ± 0.59	45.62 ± 0.59	45.41 ± 0.60	44.80 ± 0.61	−0.47 ± 0.07	−0.93 ± 0.15	−2.25 ± 0.35
			D_100%_	27.99 ± 3.96	27.88 ± 3.95	27.78 ± 3.93	27.49 ± 3.88	−0.36 ± 0.06	−0.72 ± 0.12	−1.75 ± 0.28
HX155c	11.40	0.50	D_90%_	45.93 ± 0.59	45.13 ± 0.59	44.39 ± 0.64	42.45 ± 0.76	−1.73 ± 0.27	−3.35 ± 0.51	−7.57 ± 1.05
			D_100%_	28.03 ± 3.97	28.03 ± 3.97	27.30 ± 3.85	26.37 ± 3.68	−1.35 ± 0.22	−2.61 ± 0.42	−5.90 ± 0.87
HX156c	16.46	0.26	D_90%_	40.51 ± 0.49	39.48 ± 0.52	38.59 ± 0.56	36.49 ± 0.68	−2.53 ± 0.39	−4.75 ± 0.68	−9.92 ± 1.19
			D_100%_	25.42 ± 3.42	24.93 ± 3.33	25.42 ± 3.42	23.50 ± 3.08	−1.92 ± 0.31	−3.60 ± 0.55	−7.51 ± 1.01
HX160c	6.01	5.70	D_90%_	61.73 ± 0.89	61.59 ± 0.89	61.46 ± 0.89	61.05 ± 0.90	−0.22 ± 0.04	−0.44 ± 0.07	−1.10 ± 0.17
			D_100%_	35.65 ± 5.60	35.59 ± 5.58	35.52 ± 5.57	35.65 ± 5.60	−0.18 ± 0.03	−0.36 ± 0.06	−0.90 ± 0.15
HX171c	7.02	2.30	D_90%_	56.92 ± 0.80	56.63 ± 0.80	56.35 ± 0.81	55.52 ± 0.83	−0.51 ± 0.08	−1.01 ± 0.16	−2.46 ± 0.39
			D_100%_	33.33 ± 5.10	33.19 ± 5.08	33.06 ± 5.05	32.66 ± 4.99	−0.41 ± 0.07	−0.82 ± 0.13	−2.00 ± 0.32
CaSki	5.20	3.30	D_90%_	66.91 ± 0.99	66.63 ± 0.99	66.36 ± 0.99	65.57 ± 1.01	−0.41 ± 0.07	−0.81 ± 0.13	−1.99 ± 0.32
			D_100%_	38.15 ± 6.13	38.15 ± 6.13	37.89 ± 6.08	37.51 ± 6.02	−0.34 ± 0.06	−0.68 ± 0.11	−1.67 ± 0.27
C-33A	5.60	2.70	D_90%_	64.15 ± 0.94	63.84 ± 0.94	63.54 ± 0.94	62.65 ± 0.97	−0.48 ± 0.08	−0.96 ± 0.15	−2.35 ± 0.37
			D_100%_	36.82 ± 5.84	36.67 ± 5.82	36.52 ± 5.79	36.10 ± 5.72	−0.40 ± 0.07	−0.79 ± 0.13	−1.95 ± 0.31
SiHa	6.30	2.80	D_90%_	60.17 ± 0.86	59.91 ± 0.86	59.64 ± 0.87	58.88 ± 0.88	−0.44 ± 0.07	−0.88 ± 0.14	−2.15 ± 0.34
			D_100%_	34.90 ± 5.44	34.77 ± 5.41	34.65 ± 5.39	34.28 ± 5.33	−0.36 ± 0.06	−0.72 ± 0.12	−1.76 ± 0.28
SW756	5.30	4.80	D_90%_	66.18 ± 0.97	65.99 ± 0.97	65.81 ± 0.98	65.27 ± 0.98	−0.28 ± 0.04	−0.55 ± 0.09	−1.37 ± 0.22
			D_100%_	37.80 ± 6.05	37.71 ± 6.04	37.62 ± 6.02	37.36 ± 5.98	−0.23 ± 0.04	−0.46 ± 0.08	−1.14 ± 0.18

When *α/β* = 16.46, compared with the BEDg, BED values were almost overestimated by 4.02 Gy in *D*_90%_ and 1.92 Gy in *D*_100%_ at the source activity of 2 Ci, respectively. This overestimation phenomenon further emphasizes the importance of using a more comprehensive BEDg formula for an accurate radiotherapy dose calculation.

In the nine cervix tumor cell lines, the percent values of Δ*BED* (%) were 9.92 ± 1.19 and 7.51 ± 1.01; 7.57 ± 1.05 and 5.90 ± 0.87; 2.46 ± 0.39 and 2.00 ± 0.32; 2.35 ± 0.37 and 1.95 ± 0.31; 2.25 ± 0.35 and 1.75 ± 0.28; 1.75 ± 0.28 and 1.76 ± 0.28; 1.99 ± 0.32 and 1.67 ± 0.27; 1.37 ± 0.22 and 1.14 ± 0.18 as well as 1.10 ± 0.17 and 0.90 ± 0.15 in *D*_90%_ and *D*_90%_ of HRCTV for HX156c, HX155c, HX171c, C33A, HX151c, SiHa, CaSki, SW756, and HX160c, respectively. Paired sample t- tests on the BEDg and BED values of dosimetric indicators corresponding to different Organs at Risk (OARs) and tumor cells under different radiation source activities showed statistical significance (*P* = 0.000). This is because BEDg is a monotonically decreasing function of treatment time, and the treatment time is inversely proportional to the radiation source activity. Therefore, BEDg is a monotonically decreasing function of radiation source activity. This is shown in **Table 5** and **Figure 3**.

**Table 5:** The BED and BEDg of OARs calculated with *α/β* = 3.0 and *T*_1_ *_/_*_2_ under the conditions of different source activity.

OARs	Metrics	BED	Source Activity (Ci)					
			10	5	2	10	5	2
				BEDg (Gy)			ΔBED (%)	
Bladder	D0.1*cc*	81.66 ± 23.28	79.09 ± 22.41	76.70 ± 21.60	70.44 ± 19.52	−3.06 ± 0.57	−5.91 ± 1.07	−13.37 ± 2.27
	D2*cc*	52.63 ± 13.39	51.10 ± 12.87	49.68 ± 12.38	45.97 ± 11.15	−2.80 ± 0.56	−5.41 ± 1.06	−12.23 ± 2.26
	D5*cc*	39.98 ± 11.44	38.88 ± 11.00	37.86 ± 10.59	35.20 ± 9.56	−2.61 ± 0.57	−5.05 ± 1.09	−11.40 ± 2.33
Rectum	D0.1*cc*	49.20 ± 16.90	47.79 ± 16.26	46.49 ± 15.66	43.09 ± 14.13	−2.73 ± 0.52	−5.27 ± 0.99	−11.92 ± 2.10
	D2*cc*	31.39 ± 10.89	30.59 ± 10.51	29.85 ± 10.16	27.91 ± 9.25	−2.42 ± 0.51	−4.68 ± 0.96	−10.57 ± 2.06
	D5*cc*	22.17 ± 7.23	21.66 ± 7.00	21.19 ± 6.78	19.96 ± 6.22	−2.17 ± 0.48	−4.19 ± 0.90	−9.48 ± 1.94
Sigmoid	D0.1*cc*	57.60 ± 17.14	55.89 ± 16.38	54.30 ± 15.70	50.15 ± 13.97	−2.85 ± 0.57	−5.51 ± 1.08	−12.45 ± 2.27
	D2*cc*	36.23 ± 9.03	35.27 ± 8.65	34.37 ± 8.30	32.04 ± 7.44	−2.55 ± 0.53	−4.93 ± 1.00	−11.13 ± 2.12
	D5*cc*	26.39 ± 8.57	25.74 ± 8.26	25.14 ± 7.98	23.58 ± 7.27	−2.26 ± 0.62	−4.37 ± 1.18	−9.88 ± 2.56
Intestine	D0.1*cc*	58.46 ± 28.61	56.76 ± 27.73	55.18 ± 26.90	51.05 ± 24.73	−2.73 ± 0.64	−5.27 ± 1.21	−11.91 ± 2.62
	D2*cc*	33.44 ± 15.50	32.58 ± 15.02	31.78 ± 14.57	29.69 ± 13.40	−2.39 ± 0.59	−4.61 ± 1.11	−10.41 ± 2.42
	D5*cc*	26.29 ± 11.94	25.66 ± 11.59	25.08 ± 11.26	23.55 ± 10.41	−2.22 ± 0.55	−4.28 ± 1.06	−9.68 ± 2.29

## 4 Discussion

In this study, we utilized a comprehensive and innovative approach by incorporating intrafraction DNA damage repair and cellular repopulation into our model to assess the impact of source decay on the biologically effective dose (BED) during the source-exchange cycle in cervical cancer radiotherapy. Our results unequivocally demonstrate that source decay has a profound effect on intrafraction DNA damage repair, which in turn substantially influences BED. This novel finding has great significance in the field of radiotherapy. This challenges the conventional understanding of radiation dose delivery and highlights the necessity of accounting for source decay in treatment planning. Recognizing this relationship, we can potentially enhance the accuracy of radiation therapy, leading to improved treatment outcomes in patients with cervical cancer.

Notably, we observed a positive correlation between the duration of each treatment fraction and the extent of the source decay. The effect of source decay on the Biological Effective Dose (BED) across various cervical tumor cell lines is more substantial than previously recognized. When the Ir-192 source activity decreased from 10 Ci to 2 Ci, the percentage change in BED (Δ*BED*) ranges from 0.90% ± 0.15% (for HX160c cells, *α/β* = 6.01, $T_{1/2}=5.70\;\text{h}$ ) to 14.06% ± 1.67% (for stage I and II cervical carcinoma, *α/β* = 52.63, $T_{1/2}=1.50\;\text{h}$ ). This previously underexplored relationship provides valuable insights into the practical aspects of radiotherapy. Longer treatment times owing to source decay can have far-reaching consequences for the biological effectiveness of the radiation dose. Our study is among the first to comprehensively document this relationship, which is crucial for optimizing treatment schedules and ensuring the delivery of a biologically effective dose to the tumor, while minimizing damage to normal tissues.

When comparing the BED calculated using the simplified formula with that obtained from the full-form BED formula, we found that the ΔBED increased as the source decayed. This discovery has challenged the widespread use of simplified BED formulas in clinical practice. Overestimation of BED by the simplified formula, particularly at lower source activities, can lead to inaccurate treatment planning and suboptimal patient care. Our study’s focus on this issue offers a more accurate assessment of the biological dose delivered during radiotherapy. By highlighting the limitations of the simplified formula, we contribute to the growing body of evidence supporting the adoption of more comprehensive BED calculations in cervical cancer radiotherapy, which is essential for improving the treatment efficacy and reducing the risk of treatment-related complications.

During our investigation of various cell lines, we noted that the impact of source decay on tumor proliferation was more intricate and diverse than previously thought. The α/β ratios for the numerous cell lines were distinct and smaller than the typical value of 10. This finding emphasizes the importance of considering the unique radiobiological characteristics of different tumor cell lines during radiotherapy. Tailoring radiation dosages based on these characteristics can potentially enhance the therapeutic ratio and maximize tumor control, while minimizing damage to normal tissues. Our study is one of the first to comprehensively analyze the impact of source decay on different cervical cancer cell lines, thus providing a foundation for personalized radiotherapy strategies. This personalized approach has the potential to revolutionize the treatment of cervical cancer, leading to better outcomes in patients with diverse tumor subtypes.

The conventional radiobiological parameters of TG 137 and ICRU (32), which are derived from population-averaged patient outcome studies, have limitations. Individual patients could not be accurately represented by these population-averaged values. This limitation has long been recognized; however, our study further emphasizes the need for more personalized radiobiological models. By demonstrating the significant differences in BED values among different cell lines and the impact of source decay on these values, we contribute to the growing movement towards individualized radiotherapy. Our findings suggest that personalized approaches are essential for optimizing treatment outcomes and reducing variability in patient responses to radiotherapy. Despite these imperfections, the LQ model remains a widely used tool in radiobiology (33, 34). Similar to other radiobiological modeling studies, our model’s predictions and observations were constrained by the inherent assumptions of the LQ model and DNA damage repair kinetics. Some researchers have criticized the LQ model at high-dose fractions (greater than 8 Gy–10 Gy) due to secondary biological responses, such as rapid vascular endothelial cellular apoptosis (35). However, Brenner (36) argued that the LQ model is appropriate for single fractions of up to 20 Gy. Furthermore, Shuryak et al. demonstrated that it provides similar results up to 25 Gy per fraction compared with other models (14, 37, 38).

It is important to note that our study, which calculated the BED at the prescription dose level, did not fully account for the impact of dose heterogeneity. This is a recognized limitation of the present study. While our approach was useful for identifying potential issues related to prolonged dose delivery and source decay, tumor subvolumes receiving higher doses than the prescription level may have different BED values. The reduction in BED due to prolonged dose delivery time can vary depending on the dose received by these subvolumes (39). To better understand the impact of dose heterogeneity on treatment outcomes, future studies could utilize biophysical metrics, such as effective tumor control probability, equivalent uniform dose, or equivalent uniform BED, which account for the complete dose distribution in HDR implants. Our study serves as a starting point for such investigations, highlighting the need for more comprehensive research in this area to improve the accuracy of treatment planning and patient outcomes of cervical cancer radiotherapy. In addition, HDR is typically delivered in a stepping pattern, and the rapid decay of Ir-192 indicates that most of the significant dose is accumulated over a relatively short period, despite an overall treatment time of 15 min–30 min. This delivery pattern may reduce the potential for intrafraction repairs.

In this study, we discuss the influence of different formulations on the biological dose under source attenuation conditions. The results show that the effect of source activity attenuation on the biological dose is very large when using the full BED formula. However, no clinical effects were observed. The clinical importance of ΔBEDg was validated by radiobiological modeling, which demonstrated its correlation with both tumor control and normal tissue toxicity. Leborgne et al. (40) found using the LQ model, cervical cancer patients with BEDg >120 Gy (α/β = 3 Gy) had a higher Grade 2 + 3 rectal complications comparing with the BEDg range from 100 Gy to 210 Gy, the risk roughly increasing 61%. When comparing the BEDg (α/β = 3Gy, $T_{1/2}=1.5\;\text{h}$ ) values of 78 Gy and 124 Gy, the central recurrence rates were 10.5% and 3.3%, respectively (decreasing by almost 67%). Further studies have confirmed that BEDg from 90 Gy to 95 Gy may have the best local tumor control probability (TCP), aligning with prior dose–response evidence (33). These findings demonstrate that Δ*BEDg* correlates with both tumor control and toxicity, validating its clinical utility as a personalized dose metric. Sharma et al. (41) conducted a retrospective study and found that the disease-free survival time was related to the source activity. This correlated with A reduction in the bioequivalent dose received at site A. In our study, the dynamic *α/β* ratio and BEDg equation incorporating intrafraction repair reduced the BED by 21% in squamous carcinoma at 2 Ci source activity. Differential impact analysis revealed distinct responses across tumor subtypes and OARs, with squamous carcinoma demonstrating the largest BED reduction. These advancements have enabled precise dose adjustment thresholds and linked dosimetric parameters to clinical outcomes.

Our study has the following limitations: 1. This was a retrospective study with a sample size. 2. No further clinical analysis was performed. Our future research will focus on the following two aspects: (1) Retrospective analysis and comparison of tumor changes and patient prognosis with different tumor lines and different radioactive source activities after brachytherapy. (2) A prospective study design that incorporates both radiobiological modeling and clinical outcome analysis would be valuable for further validating the impact of source decay on treatment efficacy. Such studies should aim to collect detailed clinical outcome data, including local control rates and toxicity profiles, across different source decay stages, to provide a more comprehensive understanding of this phenomenon.

## 5 Conclusion

Overall, this study offers novel insights into cervical cancer radiotherapy. Source decay significantly affected DNA damage repair and BED values in Ir-192 HDR BT for cervical cancer. This finding is crucial, as it emphasizes the need to consider source decay in treatment planning, which can enhance treatment precision and patient outcomes. By employing the full-form BED formula (BEDg), which accounts for intrafraction repair and source activity variations, we demonstrate that the simplified BED equation significantly overestimates the BED, compared to those derived from the simplified BED formula. Our findings reveal substantial BED variations across different cervical cancer cell lines and normal tissues, with maximum Δ*BED* values reaching up to 14.06% ± 1.67% for tumor HRCTV (*α/β* = 10, stages *I* and *II*) and 13.37% ± 2.27% for organs at risk (e.g., bladder, *α/β* = 3) as source activity decays to 2 Ci. Source attenuation affects different cervix tumor cell lines differently, with BED percentage values ranging from 0.90 ± 0.15 to 14.06 ± 1.67 when the source decays from 10 Ci to 2 Ci. This shows the importance of personalized radiotherapy based on cell line characteristics, which is a significant innovation in the field. When the source activity is low, we recommend using more comprehensive models such as the full-form BED equation. This can improve the dose evaluation and treatment planning accuracy, guiding future research and clinical decisions in cervical cancer radiotherapy.

## Data Availability

The raw data supporting the conclusions of this article will be made available by the authors, without undue reservation.
